# Spatial, temporal, and spatiotemporal analysis of malaria in Hubei Province, China from 2004–2011

**DOI:** 10.1186/s12936-015-0650-2

**Published:** 2015-04-08

**Authors:** Jing Xia, Shunxiang Cai, Huaxun Zhang, Wen Lin, Yunzhou Fan, Juan Qiu, Liqian Sun, Bianrong Chang, Zhijie Zhang, Shaofa Nie

**Affiliations:** Department of Epidemiology and Health Statistics, School of Public Health, Tongji Medical College, Huazhong University of Science and Technology, 430079 Wuhan, China; Institute of parasitic disease Control, Hubei Provincial Center for Disease Control and Prevention, 430079 Wuhan, China; Key Laboratory of Monitoring and Estimate for Environment and Disaster of Hubei Province, Institute of Geodesy and Geophysics, Chinese Academy of Sciences, 430077 Wuhan, China; University of Chinese Academy of Sciences, 100049 Beijing, China; Department of Epidemiology and Biostatistics, School of Public Health, Fudan University, Shanghai, P R China; Laboratory for Spatial Analysis and Modelling, School of Public Health, Fudan University, 200032 Shanghai, China

**Keywords:** Spatial, Temporal, Spatiotemporal, Analysis, Malaria

## Abstract

**Background:**

Malaria remains a public health concern in Hubei Province despite the significant decrease in malaria incidence over the past decades. Furthermore, history reveals that malaria transmission is unstable and prone to local outbreaks in Hubei Province. Thus, understanding spatial, temporal, and spatiotemporal distribution of malaria is needed for the effective control and elimination of this disease in Hubei Province.

**Methods:**

Annual malaria incidence at the county level was calculated using the malaria cases reported from 2004 to 2011 in Hubei Province. Geographical information system (GIS) and spatial scan statistic method were used to identify spatial clusters of malaria cases at the county level. Pure retrospective temporal analysis scanning was performed to detect the temporal clusters of malaria cases with high rates using the discrete Poisson model. The space-time cluster was detected with high rates through the retrospective space-time analysis scanning using the discrete Poisson model.

**Results:**

The overall malaria incidence decreased to a low level from 2004 to 2011. The purely spatial cluster of malaria cases from 2004 to 2011 showed that the disease was not randomly distributed in the study area. A total of 11 high-risk counties were determined through Local Moran’s *I* analysis from 2004 to 2011. The method of spatial scan statistics identified different 11 significant spatial clusters between 2004 and 2011. The space-time clustering analysis determined that the most likely cluster included 13 counties, and the time frame was from April 2004 to November 2007.

**Conclusions:**

The GIS application and scan statistical technique can provide means to detect spatial, temporal, and spatiotemporal distribution of malaria, as well as to identify malaria high-risk areas. This study could be helpful in prioritizing resource assignment in high-risk areas for future malaria control and elimination.

## Background

Malaria remains one of the most severe infectious diseases worldwide, it was estimated that about 3.4 billion people were at risk of malaria in 2012. There were about 207 million malaria cases worldwide, 627 000 malaria deaths are estimated to have occurred in 2012 [1]. Malaria is one of the major parasitic diseases that is widely distributed in China [2], with both *Plasmodium falciparum* and *Plasmodium vivax* historically prevalent [3]. Despite national control efforts against malaria and international support in the past decades, 26,825 malaria cases were reported from 2009 to 2011 in China [4-6].

Hubei Province is located in Central China and the middle reaches of Yangtze River. Historically, malaria transmission is unstable and prone to outbreak. The main malaria vectors are *Anopheles sinensis* and *Anopheles anthropophagus*. In Hubei Province, 18 counties are within the *A. sinensis* and *A. anthropophagus* distribution areas, whereas 84 counties are within *A. sinensis* areas [7]. Two peak epidemics occurred in 1954 and 1970, with annual incidences as high as 2,098/100,000 population and 6,024/100,000 population, respectively [8]. *Plasmodium vivax, P. falciparum*, and *P.malariae* were endemic in Hubei, with *P. vivax* as the dominant species. Indigenous *P. falciparum* and *P. malariae* were successfully eliminated in 1963 [9]. Through comprehensive implementation of effective interventions, malaria incidence started decreasing in 1980 and further decreased from 898/100,000 population in 1981 to 3/100,000 population in 2001 [10]. However, malaria has resurged in 2002, occurring at 9/100,000 population [11]. The incidence of malaria has steadily decreased since 2004, but remains relatively high in several counties [9,12].

A National Malaria Control Programme of 2006–2015 was formulated by the Ministry of Health in 2006 and, in 2010, the Chinese government launched the National Malaria Elimination Programme (NMEP) [3,13]; the Hubei Province aims to eliminate local malaria by 2015. A better understanding of spatial, temporal, and spatiotemporal distribution of malaria would help in identifying the high-risk areas and periods of malaria for the effective control and elimination malaria in Hubei Province.

Spatial-temporal epidemiology of malaria has provided us a useful method to understanding spatial and temporal patterns of malaria epidemics, assessing changes in malaria transmission and identifying malaria epidemics areas and periods with a higher risk at different scale [14-18]. A geographic information system (GIS) with spatial statistics, spatial scan and spatial-temporal scan has been widely applied to recognize the spatial and temporal variation features of malaria [19-21].

In this study, the space, time, and space-time clusters of malaria cases were investigated at the country level in Hubei Province between 2004 and 2011. Two cluster detection methods were used to identify the malaria spatial cluster in 2004–2011.

## Methods

### Study area

**Figure 1:**
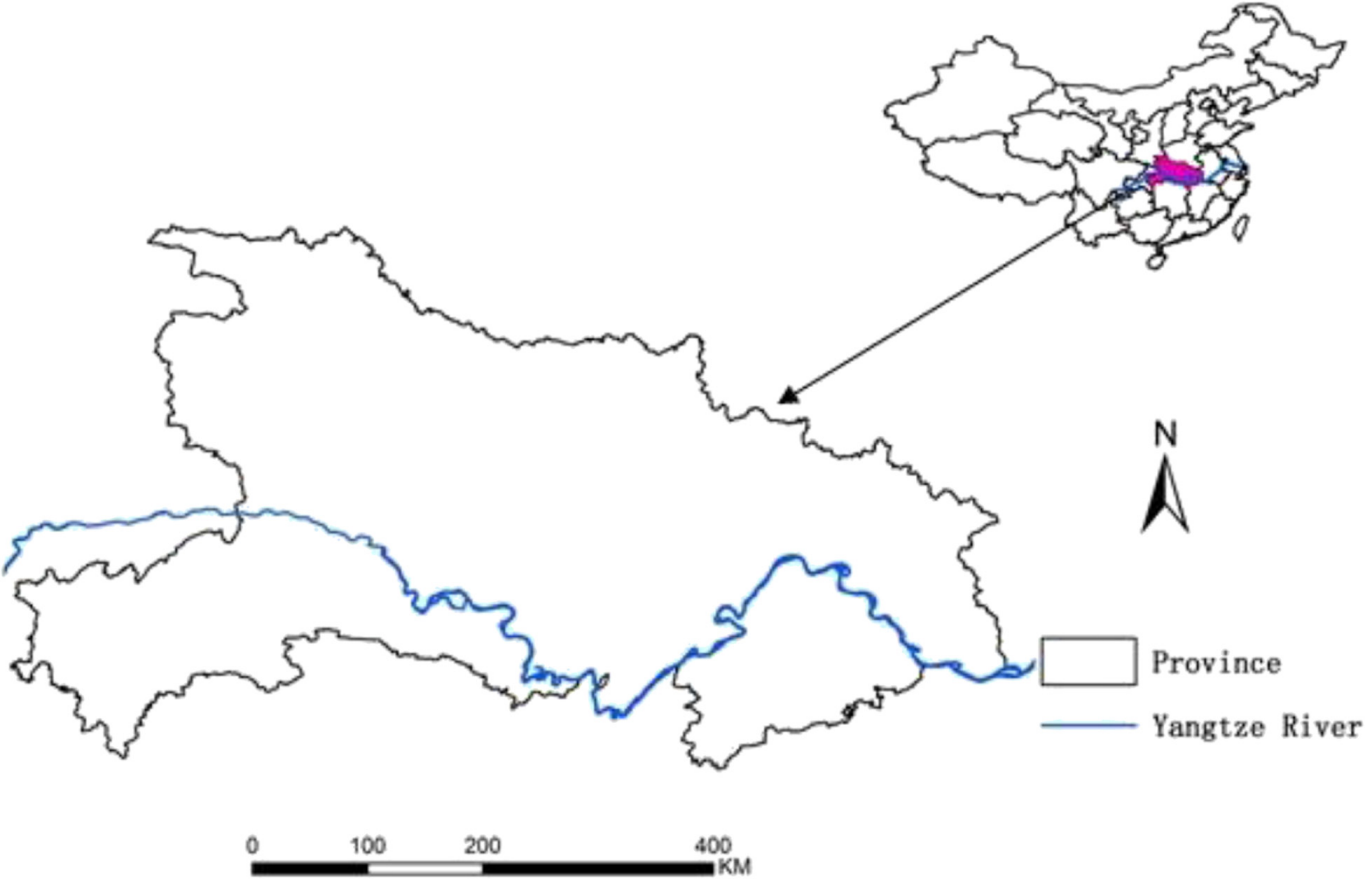
**Location of Hubei Province, China.**

The study site is Hubei Province (108°21′ ~ 116°07′E, 29°05′ ~ 33°20′N), which is located in the middle and lower reaches of Yangtze River. Subtropical monsoon climate and high rainfall occurrence comprise an ideal environment for malaria transmission. The maximum distance from east to west is approximately 740 km, and that from north to south is approximately 470 km. The area has a population of 57,237,740 (sixth national census in 2010) and encompasses 185,900 km^2^ (Figure 1).

### Malaria data

Data on malaria cases at the county in Hubei Province level between 2004 and 2011 were obtained from the China Information System for Disease Control and Prevention. The information system requires that malaria must be reported online. The malaria cases were identified on the basis of the national standard regimen. The data, which include the province name, county name, age, gender, and places where the infection was diagnosed and acquired, were also defined. The retrospective analysis of local malaria cases in Hubei Province from 2004 to 2011 was then performed.

### GIS-mapping and global spatial autocorrelation analysis

The annual malaria incidence per 100,000 individuals at each county was determined for the past eight years. The malaria incidence at county level in Hubei Province was plotted using GIS mapping technique by year. The annual malaria incidence at the county level for each year was determined to assess the malaria spatial distribution. The global spatial autocorrelation was investigated using Global Moran’s *I* statistics in ArcGIS software version 10.0 (ESRI Inc., Redlands, CA). Moran’s *I* statistical analysis tests the null hypothesis that measures the values at a location independent of values at other locations. The values vary from −1 to 1. Positive (negative) values indicate the presence of positive (negative) spatial autocorrelation, whereas a zero value indicates a random spatial pattern [22].

### Spatial cluster analysis

The spatial clustering of malaria was performed by using two cluster detection methods to identify the high-risk areas. The first method is Anselin’s Local Moran’s *I* in ArcGIS software [23]. Anselin’s Local Moran’s *I* [24] enabled the detection of the spatial autocorrelation for a county with its adjacent county. The weight element was assumed as 1 if two counties are neighbors; otherwise, we assumed the value as 0 in the weight matrix which defines the spatial autocorrelation among counties. Spatial clusters of malaria are identified by detecting local areas where high incidence counties border other high incidence counties (high-high pattern) and where high counties border low incidence counties (high-low pattern).

The second method was Kulldorff’s spatial scan statistical analysis [25,26] implemented in SaTScan software (version 8.0, Kulldorff and Information Management Services, Inc.). SaTScan imposes circular windows of varying sizes on the spatial data to detect high-risk spatial clusters of cases. In this study, the maximum spatial cluster size of the population at risk was set to 20%. The observed cases were compared with expected cases inside and outside each window, and the risk ratios were estimates on the basis of Poisson distribution. With the use of circular scanning windows, the cluster statistical significance was investigated with a log likelihood ratio test using the number of Monte Carlo replication sets, which was set to 999, under the null hypothesis of random distribution. The method is used to identify not only the most significant cluster, but also a number of secondary potential clusters.

The frequency of spatial cluster occurrence was calculated for each county from 2004 to 2011. The frequencies were calculated considering either (i) clusters detected by one of the two cluster detection methods above (weak evidence of clustering) or (ii) clusters detected by both two cluster detection methods above (strong evidence of clustering).

### Temporal and space-time cluster analysis

Pure retrospective temporal analysis scanning was performed by software to detect the temporal clusters of malaria cases with high rates using the discrete Poisson model. In this study, the time aggregation length was set to one month, and the maximum temporal cluster size was set to the default value of ≤ 50% within the study period. For each window of varying position and size, the software estimated the risk of malaria inside and outside each window, and the null hypothesis of equal risk. The space-time cluster was detected with high rates through the retrospective space-time analysis scanning using the discrete Poisson model. The space-time scan statistic was defined by a cylindrical window with a circular (or elliptic) geographic base, the height of which corresponds to time.

## Results

### Malaria incidence and global spatial autocorrelation of malaria in Hubei Province

**Figure 2:**
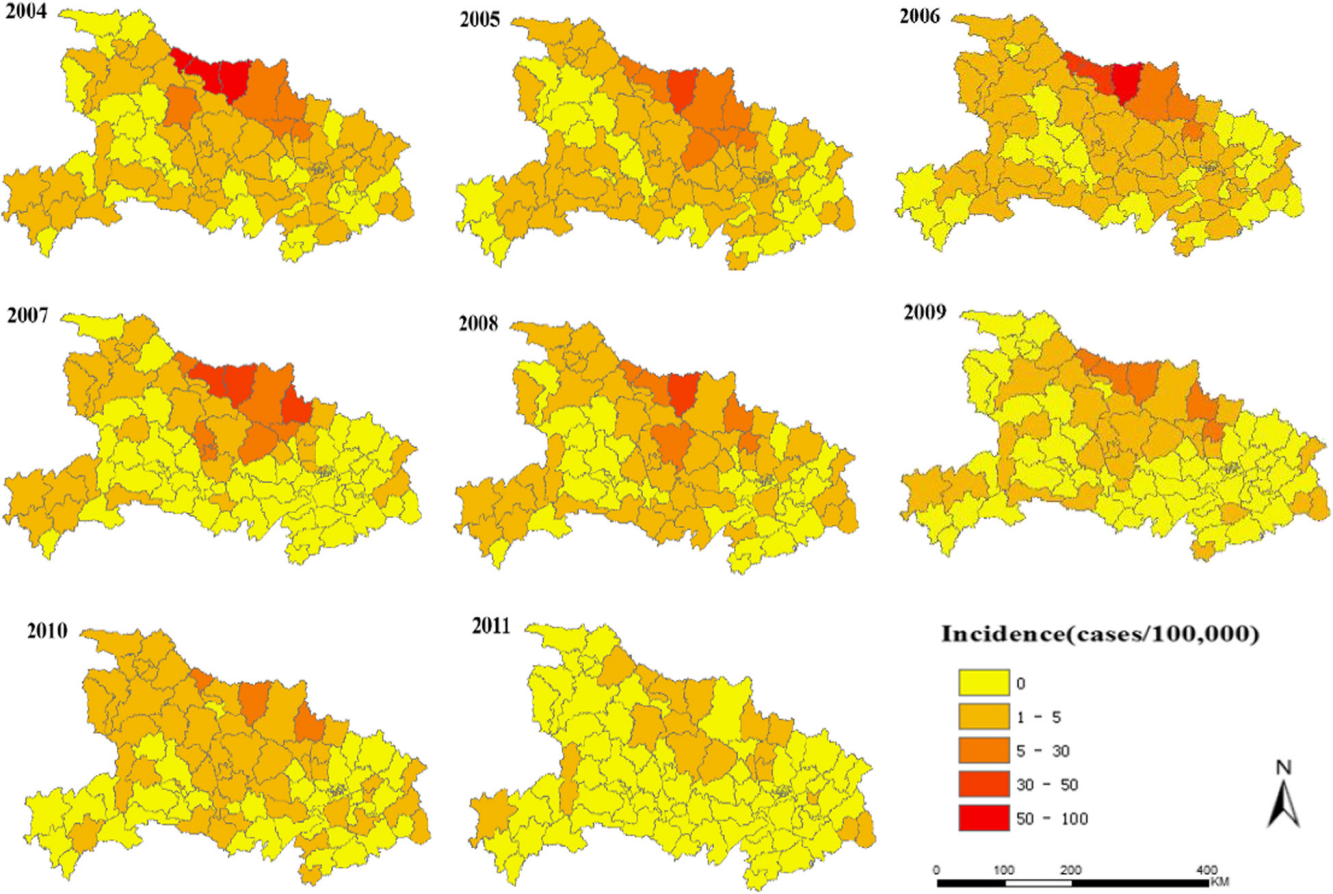
**Annual malaria incidence at county level in Hubei Province from 2004 to 2011.**

In Hubei Province, a total of 9,840 malaria cases have been reported from 2004 to 2011. Figure 2 shows the annual malaria incidence from 2004 to 2011. Table 1 shows that the number of counties with reported malaria cases decreased from 67 in 2004 to 19 in 2011, and the malaria incidence decreased from 4.40/100,000 individuals in 2004 to 0.14/100,000 individuals in 2011. Malaria incidence per year steadily decreased despite the slight resurgence in 2006. The global spatial autocorrelation analysis for the annual malaria incidence in Hubei Province showed that Moran’s *I* values were statistically significant for each year, indicating a significant overall spatial autocorrelation.

**Table 1:** **Incidence (cases/100,000) of malaria and its global spatial autocorrelation in Hubei Province, China**

**Year**	**N**	**Incidence rate**	***I***	***Z***	**p-value**
2004	67	4.40	0.17	4.75	0.00
2005	66	2.66	0.25	6.19	0.00
2006	63	3.06	0.19	5.21	0.00
2007	65	3.02	0.20	5.05	0.00
2008	55	1.87	0.21	5.46	0.00
2009	54	1.19	0.17	4.50	0.00
2010	52	0.68	0.17	4.24	0.00
2011	19	0.14	0.07	2.10	0.04

N: number of counties reporting malaria case.

I: the Global Moran’s *I* coefficient.

Z: the Global Moran’s *I* statistic value.

P: p-value for the Global Moran’s *I* statistic.

### Distribution of malaria spatial clustering

**Figure 3:**
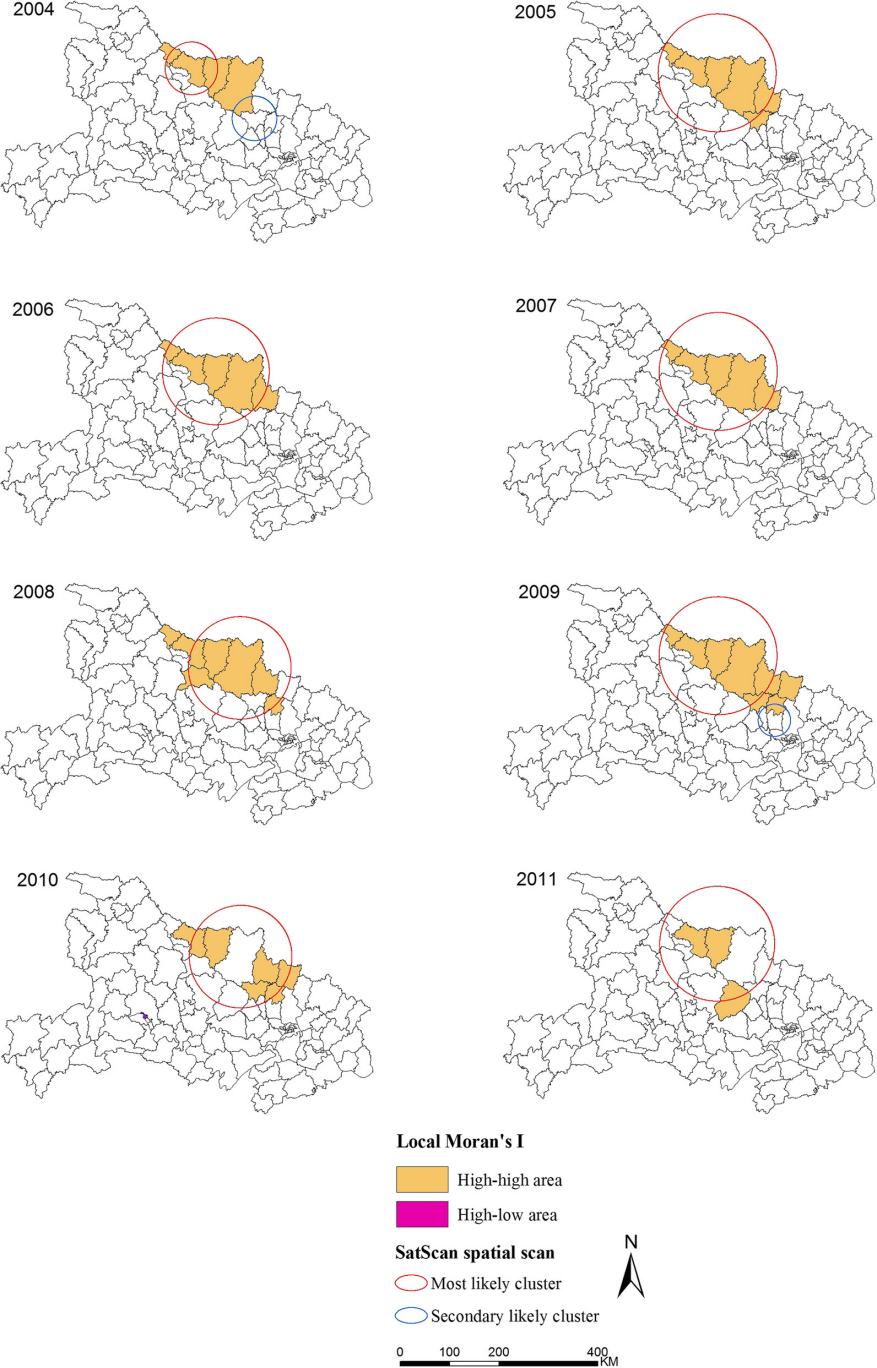
**Annual spatial clusters of malaria cases identified from 2004 to 2011.** Each panel shows the results of both methods. One method is the Anselin’s Local Moran’s *I* test; another is the Kulldorff’s spatial scan statistic.

The analysis of the spatial cluster of malaria cases from 2004 to 2011 showed that malaria was not randomly distributed in the study area (Table 2 and Figure 3). Figure 3 shows the results of spatial cluster analysis. These results were obtained through Local Moran’s *I* and Kulldorff’s spatial scan statistic. Ten high-high counties were identified using Local Moran’s *I* analysis from 2004 to 2011, and a high-low county was identified in 2010. The median annual malaria incidence of high-risk counties was 58.81/100,000 individuals in 2004. This value steadily decreased since 2008. In 2011, three high-high counties were identified using Local Moran’s *I* analysis, a median malaria incidence of 0.79/100,000 individuals was determined in 2011 (Table 3).

**Table 2:** **The cluster of malaria cases detected using the purely spatial clustering**

**Year**	**Type**	**N**	**Coordinates/Radius**	**Observed cases**	**Expected cases**	**RR**	**LLR**	**p-value**
2004	A	5	32.1727 N,112.2313E/53.23 km	1917	167.04	39.03	3785.83	0.00
	B	5	31.3004 N,113.6210E/45.28 km	288	150.53	2.02	53.25	0.00
2005	A	13	32.0882 N,112.7601E/119.54 km	1280	254.78	27.13	1673.24	0.00
2006	A	10	32.0882 N,112.7601E/108.22 km	1476	245.29	33.44	2185.75	0.00
2007	A	13	32.0882 N,112.7601E/119.54 km	1509	290.03	35.40	2085.96	0.00
2008	A	10	31.8849 N,113.2816E/103.99 km	864	160.20	24.28	1153.57	0.00
2009	A	13	32.0882 N,112,7601E/119.54 km	514	115.20	15.12	564.95	0.00
	B	4	30.9408 N,114.0040E/32.82 km	61	27.37	2.35	16.13	0.00
2010	A	10	31.8849 N, 113.2816 E/103.99 km	259	58.88	11.12	262.21	0.00
	B	1	30.7392 N,111.2928E/0.00 km	13	2.75	4.86	10.08	0.00
2011	A	11	32.0882 N,112.7601E/116.40 km	70	11.94	39.90	104.62	0.00

Type: A: the most likely cluster; B: secondary cluster.

N: the cluster number of county was identified by Kulldorff’s spatial scan.

RR: Relative risk; LLR: Log likelihood ratio.

**Table 3:** **Incidence (cases/100,000) of malaria of high-risk counties for each year**

**Year**	**N**	**Min**	**Q1**	**Median**	**Q3**	**Max**
2004	4	7.35	19.34	58.81	84.82	92.32
2005	6	6.90	8.24	20.50	31.23	37.55
2006	5	5.31	13.23	31.33	45.30	56.25
2007	5	5.06	16.60	31.81	37.49	41.96
2008	7	3.10	3.42	12.51	17.83	30.83
2009	8	1.61	2.44	6.05	10.81	17.69
2010	7	2.22	2.45	3.02	5.08	9.05
2011	3	0.44	0.44	0.79	2.99	2.99

N: the number of high-risk counties was identified by Local Moran’s *I*.

From 2004 to 2011, Kulldorff’s spatial scan method identified 11 different significant spatial clusters (eight most likely clusters and three secondary likely clusters), with an annual cluster number ranging from one to two. The number of counties per most likely cluster ranged from 5 to 13 from 2004 to 2011 (Table 2). In 2011, the most likely cluster, including 11 counties, was identified through Kulldorff’s spatial scan.

**Figure 4:**
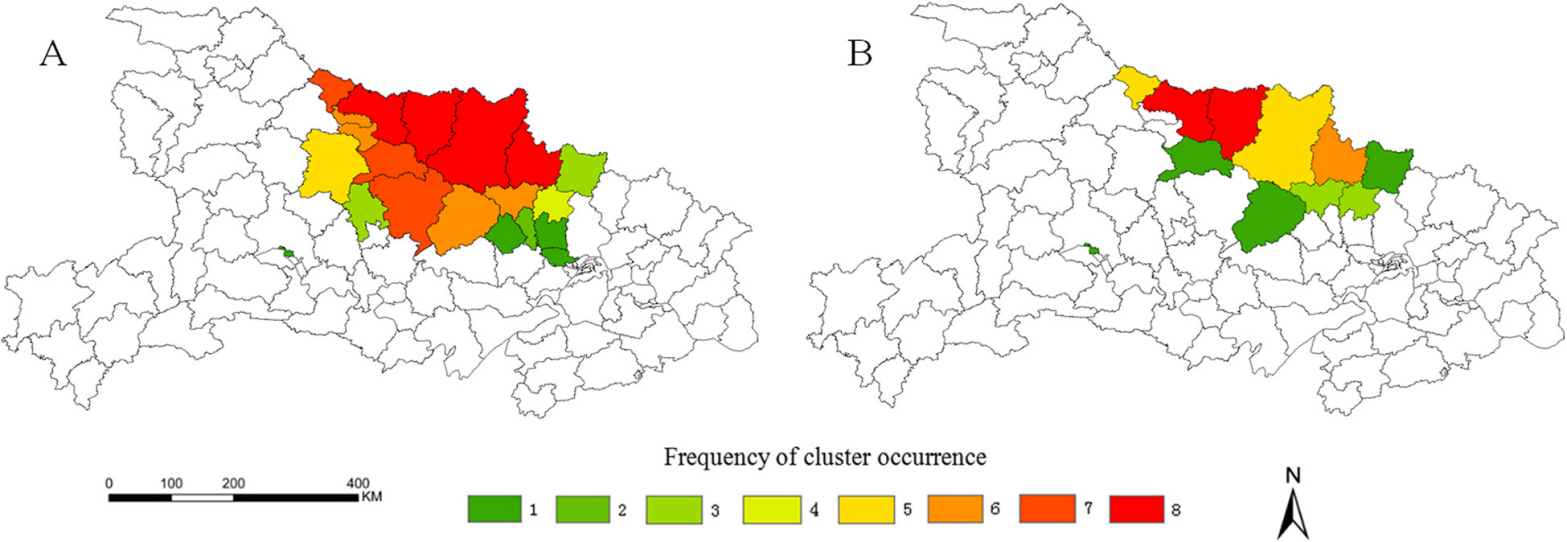
**Frequency of cluster occurrence from 2004 to 2011. A**: Frequencies of spatial cluster occurrence detected by one of the two methods (Local Moran’s *I* test or spatial scan statistic). **B**: Frequencies of spatial cluster occurrence detected by both methods.

12 counties were detected five or more times by one of the two cluster detection methods (Guangshui, Xiangzhou, Zaoyang, Yicheng, Zhongxiang, Zengdu, Anlu, Fancheng, Jingshan, Laohekou, Xiangcheng and Nanzhang), the median frequency was 6 (range = 1–8) for clusters occurrence detected by one method (Figure 4A). Clusters were found in 5 counties (Xiangzhou, Zaoyang. Zengdu, Guangshui and Laohekou) five or more times by both methods, the median frequency was 3 (range = 1–8) for clusters detected by both methods (Figure 4B).

### Distribution of malaria temporal clustering

Table 4 shows the purely temporal clusters. The temporal clusters of malaria cases in Hubei Province included four months in 2004 to 2006 (June to September), five months in 2007 to 2010 (May to September), and five months in 2011 (February to June).

**Table 4:** **The cluster of malaria cases detected using the purely temporal clustering**

**Year**	**Cluster time frame**	**Observed cases**	**Expected cases**	**RR**	**LLR**	**p-value**
2004	2004/6/1-2004/9/30	2184	882.00	9.45	1361.30	0.00
2005	2005/6/1-2005/9/30	1060	505.72	4.66	422.45	0.00
2006	2006/6/1-2006/9/30	1190	583.59	4.26	437.84	0.00
2007	2007/5/1-2007/9/30	1258	720.57	3.78	344.75	0.00
2008	2008/5/1-2008/9/30	699	445.20	2.66	122.54	0.00
2009	2009/5/1-2009/9/30	436	285.46	2.47	67.31	0.00
2010	2010/5/1-2010/9/30	228	163.48	1.95	21.54	0.00
2011	2011/2/1-2011/6/30	55	32.88	3.15	12.45	0.00

RR: Relative risk; LLR: Log likelihood ratio.

### Distribution of malaria spatiotemporal clustering

**Figure 5:**
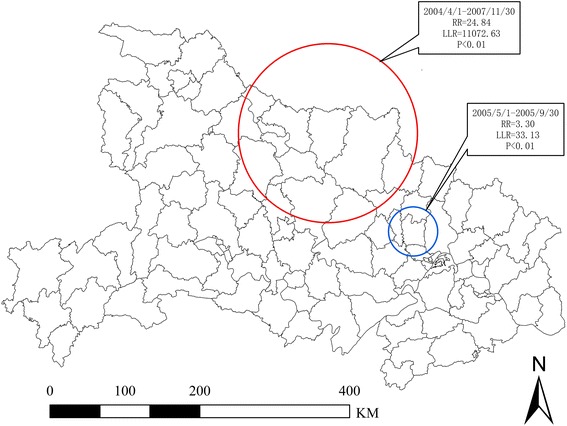
**Locations of the detected clusters of malaria cases by the space-time analysis during 2004–2011.**

Table 5 shows the cluster of malaria cases detected through the space-time scan statistics from 2004 to 2011 in Hubei Province. The most likely cluster and a secondary cluster were identified (Table 5 and Figure 5). The most likely cluster included 13 counties, and the time frame was from April 2004 to November 2007. The secondary cluster included four counties from May to September 2005.

**Table 5:** **The cluster of malaria cases detected using the retrospective space-time analysis from 2004 to 2011 in Hubei Province**

**Type**	**N**	**Time frame**	**Coordinates/Radius**	**Observed cases**	**Expected cases**	**RR**	**LLR**	**p-value**
A	13	2004/4/1-2007/11/30	32.0882 N, 112.7601 E/119.54 km	6654	763.28	24.84	11072.63	0.00
B	4	2005/5/1-2005/9/30	30.9408 N, 114.0040 E/32.82 km	67	20.42	3.30	33.13	0.00

Type: A: the most likely cluster; B: secondary cluster.

N: the cluster number of county was detected by retrospective space-time analysis.

RR: Relative risk; LLR: Log likelihood ratio.

## Discussion

GIS and scan statistical method were used to examine the changing patterns and clusters between 2004 and 2011 in Hubei Province. In this study, the results showed significant global spatial autocorrelations of malaria incidence from 2004 to 2011 (Table 1), indicating that the malaria spatial distribution still followed a clustered pattern. The overall malaria incidence has decreased to a low level from 2004 to 2011.

The spatial cluster analysis indicated that the malaria incidence significantly decreased, and the median annual malaria incidence of high-risk counties decreased from 58.81/100,000 individuals in 2004 to 0.79/100,000 individuals in 2011. In 2011, three high-risk counties were identified through Local Moran’s *I* analysis (Figure 3 and Table 3), and the spatial extent of the malaria cases remained scattered in 2011 (Figure 3 and Table 2), indicating that a large population was still at risk.

In this study, the high-risk areas from 2004 to 2011 were mainly in the *A. sinensis* and *A. anthropophagus* epidemic areas, and spatial clusters more frequently occurred within 4 counties (Xiangzhou, Zaoyang. Zengdu, Guangshui) in the *A. sinensis* and *A. anthropophagus* distribution areas and within 1 county (Laohekou) within the *A. sinensis* epidemic areas. The average vector capacity of *A. anthrophagus* was 44.47 times higher than that of *A. sinensis* [27,28], which frequently reduces the local outbreak in *A. sinensis* and *A. anthropophagus* epidemic areas [7,10]. In addition, Laohekou is more frequently detected spatial cluster area, the malaria incidence of which was higher than that in other counties within *A. sinensis* epidemic areas. This observation may be attributed to the fact that Laohekou is near the *A. sinensis* and *A. anthropophagus* epidemic areas, and the people in Laohekou have the habit of sleeping in open areas without protection during summer. Several studies on insecticide resistance have shown that *A. sinensis* was highly resistant to both deltamethrin and DDT in several parts of these areas [29,30]. The high-risk areas have always been the focus of malaria control and elimination in Hubei Province [7,9,10,12]. The corresponding effective control strategies, such as the proper use of insecticides and environmental management, are important for the malaria vector control and should therefore be implemented.

The purely temporal cluster analysis showed the high-risk months of malaria for each year from 2004 to 2011. The temporal clusters per year from 2004 to 2010 were consistent with the malaria epidemic season in Hubei Province, and the favourable climax condition in high-risk months is prone to malaria transmission [9,12]. However, the temporal cluster in 2011 was observed before the epidemic season, most likely because of the significant decrease in malaria cases caused by the effective malaria control measures implemented. A total of 80 malaria cases were reported malaria in Hubei Province in 2011 [12], offsetting the effects induced by weather factors.

Space-time clustering identified two likely clusters of malaria before December 2007. The cluster areas were high malaria incidence counties, which were mainly within *A. sinensis* and *A. anthropophagus* epidemic areas. The malaria cases in *A. sinensis* and *A. anthropophagus* epidemic areas accounted for more than 80% of the total cases in Hubei Province [7,9]. Since December 2007, the malaria cluster has not been identified because of the malaria programme of China Global Fund’s first and fifth round and malaria programme of the National Strategy Application implemented in Hubei from 2003 to 2011 [31,32]. The measures of these programmes were mainly diagnostic, standard treatment, effective prevention, health education and promotion of malaria for high-risk population.

The NMEP aims to eliminate local malaria by 2015 in Hubei Province. Thus, effective control measures are necessary to achieve this aim. This study showed that the spatial extent of malaria cases remains scattered, and high-risk areas still exist. Furthermore, imported malaria cases pose constant challenges in eliminating malaria in Hubei Province, and the percentage of foreign malaria strains increases every year [33,34]. The malaria outbreaks originating from foreign cases have been reported in non-endemic malaria areas [35-37]. Hubei Province was historically a high-endemic area, and the malaria vector remains widely distributed throughout the province [28]. This phenomenon suggests that significant attention must be directed toward high-risk areas to prevent the potential malaria resurgence similar to what occurred in Central China [38,39]. Meanwhile, malaria elimination strategies should focus on high-risk areas considering the limited resources.

In this study, two cluster detection methods were used to identify the different types of malaria clusters, as recommended and applied by other researchers [40-42]. Local Moran’s *I* analysis based on spatial attributes reflects the severity of malaria, and is biased when population sizes at risk in each county varies substantially [43]. Kulldorff’s statistical analysis detects malaria spatial distribution, focuses on the extent of malaria, and is useful in detecting circular shapes; however, it has limitations in detecting irregular circular shapes [44]. The two cluster methods can jointly improve our overall understanding of the complicated malaria spatial pattern [40].

## Conclusions

This study analysed the spatial, temporal, and space-time clusters of malaria cases at county level in Hubei Province from 2004 and 2011. In summary, the malaria incidence generally decreased to a low level from 2004 to 2011. The purely spatial and purely temporal clusters were statistically significant for each year. The malaria spatial extent remains scattered, and a large percentage of the population is still at risk. High-risk areas still exist. The study could be helpful in prioritizing the resource assignment for malaria cases in high-risk areas for more effective disease control. Such assignment must be targeted to eliminate malaria in Hubei Province.

## References

[1] WHO (2013). World Malaria Report 2013.

[2] Zhang WY, Wang LP, Fang LQ, Ma JQ, Xu YF, Jiang JF (2008). Spatial analysis of malaria in Anhui province, China. Malar J.

[3] Yin JH, Yang MN, Zhou SS, Wang Y, Feng J, Xia ZG (2013). Changing malaria transmission and implications in China towards national malaria elimination programme between 2010 and 2012. PLoS One.

[4] Zhou SS, Wang Y, Xia ZG (2011). [Malaria situation in the People’s Republic of China in 2009] (in Chinese). Chinese J Parasitology Parasitic Dis.

[5] Zhou SS, Wang Y, Li Y (2011). [Malaria situation in the People’s Republic of China in 2010] (in Chinese). Chinese J Parasitology Parasitic Dis.

[6] Xia ZG, Yang MN, Zhou SS (2012). [Malaria situation in the People’s Republic of China in 2011] (in Chinese). Chinese J Parasitology Parasitic Dis.

[7] Yuan FY, Huang GQ, Zhang HX, Pei SJ, Liu JY, Hu LQ (2010). [Status of malaria epidemic and feasibility of malaria elimination in Hubei] (in Chinese). J Pub Health Prev Med.

[8] Zhang HX, Huang GQ, Chen GY (1999). [Analysis of epidemic posture of malaria from 1971 to 1997 in Hubei Province] (in Chinese). Chinese J Vector Biol Cont.

[9] Huang GQ, Yuan FY, Hu LQ, Lin W, Zhang HX, Liu JY (2010). [Analyses of malaria epidemic situation in Hubei in 2007–2009] (in Chinese).. J Tropical Med.

[10] Pei SJ, Huang GQ, Gui AF, Zuo SL, Chen GY, Hu LQ (2004). [Analysis of malaria epidemic situation in the past ten yesrs in Hubei Province] (in Chinese). J Public Health Prev Med.

[11] Zuo SL, Chen GY, Hu LQ, Gui AF, Pei SJ, Liu JY (2004). [Analysis of malaria situation in Hubei 2003] (in Chinese). J Pub Health Prev Med.

[12] Lin W, Mao ZF (2012). [Analysis of malaria epidemic in Hubei Province (2009–2011)] (in Chinese). J Pub Health Prev Med.

[13] National Malaria Control Programme 2006–2015 [http://www.nhfpc.gov.cn/zhuzhan/zcjd/201304/34c8353e42bb4678a9cdc4f5dfc7e2f6.shtml]

[14] Coleman M, Coleman M, Mabuza AM, Kok G, Coetzee M, Durrheim DN (2009). Using the SaTScan method to detect local malaria clusters for guiding malaria control programmes. Malar J.

[15] Bautista CT, Chan AS, Ryan JR, Calampa C, Roper MH, Hightower AW (2006). Epidemiology and spatial analysis of malaria in the Northern Peruvian Amazon. Am J Trop Med Hyg.

[16] Ernst KC, Adoka SO, Kowuor DO, Wilson ML, John CC (2006). Malaria hotspot areas in a highland Kenya site are consistent in epidemic and non-epidemic years and are associated with ecological factors. Malar J.

[17] Alemu K, Worku A, Berhane Y, Kumie A (2014). Spatiotemporal clusters of malaria cases at village level, northwest Ethiopia. Malar J.

[18] Gaudart J, Poudiougou B, Dicko A, Ranque S, Toure O, Sagara I (2006). Space-time clustering of childhood malaria at the household level: a dynamic cohort in a Mali village. BMC Public Health.

[19] Zhou G, Sirichaisinthop J, Sattabongkot J, Jones J, Bjornstad ON, Yan G (2005). Spatio-temporal distribution of *Plasmodium falciparum* and *P. vivax* malaria in Thailand. Am J Trop Med Hyg.

[20] Ndiath M, Faye B, Cisse B, Ndiaye JL, Gomis JF, Dia AT (2014). Identifying malaria hotspots in Keur Soce health and demographic surveillance site in context of low transmission. Malar J.

[21] Hui FM, Xu B, Chen ZW, Cheng X, Liang L, Huang HB (2009). Spatio-temporal distribution of malaria in Yunnan Province, China. Am J Trop Med Hyg.

[22] Moran P (1950). Notes on continuous stochastic phenomena. Biometrika.

[23] Anselin L (1995). Local indicators of spatial association-LISA. Geogr Anal.

[24] Anselin L, Sridharan S, Gholston S (2007). Using exploratory spatial data analysis to leverage social indicator databases: the discovery of interesting patterns. Soc Indic Res.

[25] Kulldorff M, Nagarwalla N (1995). Spatial disease clusters: detection and inference. Stat Med.

[26] Kulldorff MA (1997). Spatial scan statistic. Commun Stat Theory Methods.

[27] Huang GQ, Zhang HX, Liu JY, Yuan FY, Yu PH, Chen GY (2000). [Study on the ecology character of distribution and role of malarial transmission in anopheles anthropophagus in Hubei, China] (in Chinese). Chinese Journal of Vector Biology and Control.

[28] Chen GY, Zhang HX, Huang GQ, Li SH, Yuan FY (2006). [Study on the relationship between the ecology of anopheles vectors and malaria infection in Hubei Province] (in Chinese). China Tropical Med.

[29] Pei SJ, Zhang HX, Li KJ, Hu LQ, Xia J, Shang XP (2014). [Monitoring of resistance of anopheles sinensis to deltamethrin in Hubei province, China] (in Chinese). Chinese J Vector Biol Cont.

[30] Wang DQ, Xia ZG, Zhou SS, Zhou XN, Wang RB, Zhang QF (2013). A potential threat to malaria elimination: extensive deltamethrin and DDT resistance to *Anopheles sinensis* from the malaria-endemic areas in China. Malar J.

[31] Pei SJ, Yuan FY, Huang GQ, Gui AF, Zuo SL, Chen GY (2007). [Evaluation of results in implementation of global fund on malaria control in Hubei Province in last three years] (in Chinese). China Tropical Med.

[32] Gui AF, Huang GQ, Pei SJ, Zuo SL, Chen GY, Hu LQ (2007). [Mid-term evalution on the Hubei global fund of malaria control project] (in Chinese). J Pub Health Prev Med.

[33] Li KJ, Huang GQ, Zhang HX, Lin W, Dong XR, Pi Q (2013). [Epidemic situation and control strategy of imported malaria in Hubei Province from 2006 to 2011] (in Chinese). Chinese J Schistosomiasis Cont.

[34] Huang GQ, Hu LQ, Zhang HX, Lin W, Zheng L, Li LJ (2013). [Potential infection of imported malaria and control measures in Hubei province] (in Chinese). China Tropical Med.

[35] Kruger A, Rech A, Su XZ, Tannich E (2001). Two cases of autochthonous Plasmodium falciparum malaria in Germany with evidence for local transmission by indigenous *Anopheles plumbeus*. Trop Med Int Health.

[36] Limongi JE, Chaves KM, Paula MB, Costa FC, Silva Ade A, Lopes Ide S (2008). Malaria outbreaks in a non-endemic area of Brazil, 2005. Rev Soc Bras Med Trop.

[37] Zoller T, Naucke TJ, May J, Hoffmeister B, Flick H, Williams CJ (2009). Malaria transmission in non-endemic areas: case report, review of the literature and implications for public health management. Malar J.

[38] Zhou SS, Huang F, Wang JJ, Zhang SS, Su YP, Tang LH (2010). Geographical, meteorological and vectorial factors related to malaria re-emergence in Huang-Huai River of central China. Malar J.

[39] Sleigh AC, Liu XL, Jackson S, Li P, Shang LY (1998). Resurgence of vivax malaria in Henan Province, China. Bull World Health Organ.

[40] Ward MP, Carpenter TE (2000). Techniques for analysis of disease clustering in space and in time in veterinary epidemiology. Prev Vet Med.

[41] Hu Y, Xiong C, Zhang Z, Luo C, Ward M, Gao J (2014). Dynamics of spatial clustering of schistosomiasis in the Yangtze River Valley at the end of and following the World Bank Loan Project. Parasitol Int.

[42] Hu Y, Xiong C, Zhang Z, Luo C, Cohen T, Gao J (2014). Changing patterns of spatial clustering of schistosomiasis in Southwest China between 1999–2001 and 2007–2008: assessing progress toward eradication after the World Bank Loan Project. Int J Environ Res Public Health.

[43] Gelman A, Price PN (1999). All maps of parameter estimates are misleading. Stat Med.

[44] Aamodt G, Samuelsen SO, Skrondal A (2006). A simulation study of three methods for detecting disease clusters. Int J Health Geogr.

